# Longitudinal, observational study on associations between postoperative nutritional vitamin D supplementation and clinical outcomes in esophageal cancer patients undergoing esophagectomy

**DOI:** 10.1038/srep38962

**Published:** 2016-12-13

**Authors:** Lu Wang, Cong Wang, Jiangfeng Wang, Xiaochen Huang, Yufeng Cheng

**Affiliations:** 1 Department of Radiation Oncology, Qilu Hospital of Shandong University, Jinan, Shandong, China

## Abstract

Vitamin D can exert anticancer effect beyond bone and calcium metabolism. We aimed to investigate whether postoperative vitamin D supplementation affects quality of life (QOL) and survival in esophageal cancer (EC) patients. We utilized the widely used EORTC QLQ-C30 and QLQ-OES18 to assess QOL at EC diagnosis and 24 months after surgery. Generalized estimating equations (GEEs) were used to analysis the association of vitamin D supplement use with QOL. Kaplan-Meier method and Cox regression model were used to evaluate the prognostic value of vitamin D supplementation. The notably improved QOL were found among vitamin D supplementation users compared with non-users (*p* < 0.05). Kaplan-Meier analysis revealed that vitamin D supplement use was significantly associated with improved disease-free survival (DFS) (*p* = 0.030), but not related to overall survival (OS) (*p*** = **0.303). The multivariable analysis further demonstrated vitamin D supplement use as an independent prognostic factor for DFS (*p*** = **0.040; HR 0.610; 95% CI 0.381–0.978). In conclusion, these results showed that vitamin D supplement use could serve as a promising intervention to enhancing QOL and prolonging DFS in EC.

EC is the sixth cause of cancer-related death and the eighth most common carcinoma in the world^1^. There are estimated 455,800 new EC cases and 400,200 EC-caused deaths in 2012 worldwide^2^. Surgery, as the best curative option for nonmetastatic patients, is the major treatment for it. Even though patients undergo esophagectomy, the 5-year OS rate is only 15–20%^3^. As previous studies reported, high incidence and mortality, as well as poor QOL and prognosis are well-recognized features of EC. Undoubtedly, it has already become a major public health concern in the world. As a result, there is an urgent need to improve QOL and survival in EC patients following surgery.

As is known to all, cancer patients’ QOL and survival are not only determined by tumor pathology but also by host factors, such as healthy diet^4^. An interest in dietary supplementation has been particularly popular among cancer survivors^5^, although limited existing data supported that it could contribute to positive clinical outcomes after cancer diagnosis. One widely used dietary supplementation is vitamin D. No vitamins have received more attention in recent years for their potential relationships with cancer causation and outcomes than vitamin D^6^. At present, the role of vitamin D is not only limited to regulate bone metabolism and maintain calcium homeostasis^7^. A large number of preclinical studies have demonstrated several effects of vitamin D on the hallmarks of cancer, including anti-proliferative, anti-metastasis, anti-angiogenesis, pro-apoptosis and pro-differentiation activities^8^,^9^,^10^,^11^,^12^,^13^,^14^. Observational studies have also revealed significant relationships of vitamin D with breast cancer, colorectal cancer, prostate cancer and pancreatic cancer^6^,^10^,^15^,^16^,^17^. Additionally, vitamin D deficiency could increase the risk of developing cancer and lead to a lower QOL^18^,^19^. Therefore, more and more evidence supported that vitamin D supplement use might enhance QOL and decrease risk of recurrence or mortality among cancer patients. However, no work has been done to address the role of vitamin D supplementation that plays in clinical outcomes in EC patients following surgery.

To investigate the chemopreventive potential and anticancer action of vitamin D, we conducted a longitudinal, observational study of supplementation with vitamin D for improving poor clinical outcomes in EC patients. We primarily hypothesized that vitamin D supplement use could improve QOL during EC treatment and recovery phases. Our secondary hypotheses addressed the relationships between vitamin D supplementation and the risk of recurrence or mortality in cancer patients who underwent esophagectomy.

## Results

### Patients’ characteristics

A total of 303 patients were eventually recruited in our study. 49 (16.2%) patients regularly used vitamin D supplementation after surgery, and they usually took it 200–400 IU daily over one year via self-report. However, 254 (83.8%) patients were vitamin D supplementation non-users. Additionally, there were only 181 EC survivors at 2 years of follow-up. 32 cases took regularly vitamin D supplementation among these survivors, whereas 149 did not. Patients’ baseline characteristics are listed in Table 1. There were significant differences in age (*p*** = **0.006), education (*p*** = **0.008), income (*p*** = **0.003), fat intake (*p*** = **0.020), physical activity (*p*** = **0.013), smoking (*p*** = **0.003) and history of diabetes (*p*** = **0.009) between vitamin D supplementation users group and non-users group. Besides, no significant differences were found in other characteristics (all *p* > 0.05).

### Association between vitamin D supplement use and QOL

Table 2 presents the association between postoperative vitamin D supplement use and QOL over 24 months of follow-up. After adjustment for potential confounders, which are age at diagnosis, gender, education, income, BMI, dietary habits, physical activity, sun exposure, smoking, alcohol, past medical history, tumor characteristics, type of surgery, treatment regimen and QOL at diagnosis, the significant associations were observed between vitamin D supplement use and certain aspects of QOL, including global health (*β* = −2.985, 95% CI −5.880–−0.089, *p*** = **0.043), physical functioning (*β*** = **−3.640, 95% CI −6.085–−1.196, *p* = 0.004), social functioning (*β*** = **−6.347, 95% CI −11.178–−1.516, *p*** = **0.010), fatigue (*β*** = **6.110, 95% CI 0.531–11.689, *p*** = **0.032) and appetite loss (*β*** = **10.435, 95% CI 1.107–19.763, *p*** = **0.028) measured by QLQ-C30 as well as eating (*β*** = **5.365, 95% CI 0.876–9.853, *p*** = **0.019) and trouble with taste (*β*** = **8.491, 95% CI 0.882–16.100, *p*** = **0.029) measured by QLQ-OES18.

### The prognostic value of vitamin D supplement use

**Figure 1:**
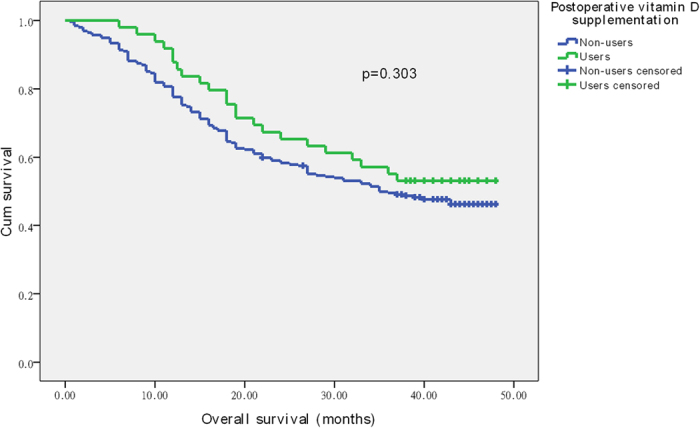
Overall survival related to postoperative vitamin D supplementation.

**Figure 2:**
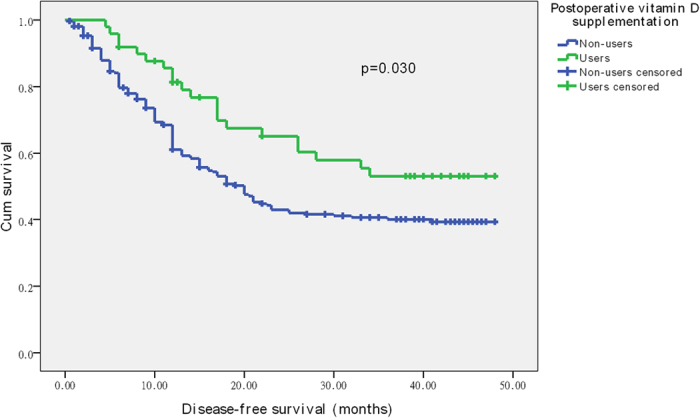
Disease-free survival related to postoperative vitamin D supplementation.

The 3-year OS and 3-year DFS associated with vitamin D supplement use, calculated by Kaplan-Meier method, are shown in Figs 1 and 2. The 3-year OS rates were 49.5% in the non-users group and 55.1% in the users group. The 3-year DFS rates were 40.1% and 53.1% in the non-users group and users group, respectively. Patients who received vitamin D supplementation were more likely to have improved DFS (*p*** = **0.030). However, no positive association of vitamin D supplement use with OS was found in our study (*p*** = **0.303).

### Univariate and multivariate analysis

The factors related to OS and DFS on univariate analysis are shown in Table 3. In univariate analysis, physical activity, pathological type, tumor length, T stage, TNM stage, lymph node metastasis, the number of lymph node metastases, positive lymph node ratio and treatment regimen (all *p* < 0.05) were associated with both OS and DFS. However, vitamin D supplementation was only associated with DFS (*p*** = **0.035; HR 0.611; 95% CI 0.386–0.966) but not related to OS (*p*** = **0.308; HR 0.795; 95% CI 0.511–1.237). The results of multivariate Cox regression analysis of the factors related to OS and DFS are shown in Table 4. It further suggested that vitamin D supplement use was an independent prognostic factor for DFS (*p*** = **0.040; HR 0.610; 95% CI 0.381–0.978). Interestingly, we found physical activity was an independent prognostic factor for both OS (*p*** = **0.004; HR 0.957; 95% CI 0.928–0.986) and DFS (*p* < 0.001; HR 0.949; 95% CI 0.922–0.978).

## Discussion

To the best of our knowledge, this is the first report on relationship between vitamin D supplement use and clinical outcomes of EC. We found a significant improvement in QOL and DFS of EC patients undergoing esophagectomy who received vitamin D supplementation during cancer treatment and recovery phases. Considering imperfect medicare system, relatively low socioeconomic status and weak health conscious in our developing countries, it was not surprising to find that the majority of patients were vitamin D non-users and only 49 patients (16.2%) self-reported to regularly receive vitamin D supplement. The dosage of it was 200–400 IU daily, which was suggested by clinician and also recommended by Chinese Nutrition Society and World Health Organization. In summary, compared to non-users, vitamin D users tended to be younger, attended some college, had a higher income, participated in more physical activity, had lower fat intake, used less tobacco and were more likely to suffer from diabetes in our study.

In view of the limited data supporting positive effect of vitamin D supplement use on QOL for cancer, our results undoubtedly confirmed and extended recently published articles. Although we followed up the patients for 3 years, the sample size of survivors who received vitamin D was too small to sufficient statistical analysis at that time. As a result, the associations between vitamin D supplement use and QOL assessed at 2 years of follow-up were observed. In our study, EC patients who were supplemented with vitamin D had higher scores of physical functioning, social functioning and global health as well as lower scores of fatigue and appetite loss via a 24-month follow-up compared with non-users. It suggested that vitamin D users were more likely to maintain physical function, have more energy and better appetite, feel less faintness as well as enjoy life. These findings were consistent with previous study^20^, which reported that stage II colorectal cancer patients who supplemented vitamin D tended to have better QOL. Another cross-sectional study indicated that vitamin D deficiency was quite common as well as associated with fatigue and poor physical and functional well-being in advanced cancer patients^19^. Therefore, it pointed to vitamin D supplementation as a potential therapy to improve cancer patients’ QOL. Although vitamin D status is unknown in the current study, vitamin D supplement use did show a significant relationship with QOL.

To date, the association of vitamin D with cancer has been reported by several reviews,^6^,^9^,^10^,^21^,^22^,^23^,^24^,^25^ which proposed it might have potential role as anticancer drug. In addition to the influence of vitamin D on cancer risk^26^,^27^,^28^, numerous reports^29^,^30^,^31^,^32^ showed that vitamin D sufficiency could improve survival in lung cancer, breast cancer, prostate cancer and follicular lymphoma. However, the studies concerning associations between vitamin D supplementation and DFS or OS were limited and contradictory. In this study, we found that vitamin D supplement use could prolong DFS but not OS of EC patients. Our results were consistent with the findings of Zeichner *et al*.^33^. They also reported that vitamin D supplementation in patients with nonmetastatic HER2^**+**^ breast cancer was associated with improved DFS but not related to OS. While, Lewis *et al*.^20^ studied the correlation of vitamin D supplementation with risk of recurrence or mortality in stage II colorectal cancer patients, no association was observed with DFS or OS. The statistical power for survival analysis may be insufficient or confounding variables could not be completely adjusted to influence the final results or measurement bias, which may in a way explain their null results. In another recent study in women in the UK, Jeffreys *et al*.^34^ found no evidence that pre-diagnostic vitamin D supplementation was associated with survival among women with breast cancer, colorectal cancer, lung cancer and gynaecologic cancer. By contrast, we studied the correlation between post-operative vitamin D supplementation after cancer diagnosis and survival in EC patients, which did show that vitamin D supplement use after surgery could reduce the risk of recurrence to improve DFS. According to numerous existing evidences, the different results might be partially explained that pre-diagnostic vitamin D supplementation was more likely to play a role in reducing cancer risk but little associated with long-term clinical outcomes, while post-diagnostic vitamin D supplement use during cancer treatment and recovery phases might have a crucial effect on long-term survival.

Additionally, we interestingly observed physical activity was an independent prognostic factor for both DFS and OS in our study. A number of articles, which have reported the inverse relation of lower physical activity with increased risk of mortality and poor survival, were consistent with our results^35^,^36^,^37^. We strongly supported a protective effect of physical activity on prognosis of EC.

In brief, vitamin D can be obtained from foods, sun exposure and supplementation. However, the quantities of vitamin D from dietary are quite small in our country. What’s more, cancer patients are more likely to spend relatively large amounts of time indoors because of physical weakness and severe symptoms. It leads to less sun exposure contrasted with healthy people. Therefore, compared to other sources, the crucial role of vitamin D supplementation in cancer prevention cannot be ignored. Furthermore, it is worth mentioning that in addition to the well-known classic metabolic pathway, recently published articles reported that novel pathway of vitamin D metabolism *in vivo* can be initiated by CYP11A1 and modified by CYP27B1 to generate previously unrecognized vitamin D-hydroxyderivatives, which are different from 25-hydroxyvitamin D [25(OH)D] and 1,25(OH)_2_D^38^,^39^,^40^. An amount of studies have revealed that they can not only regulate bone mineralization and calcium homeostasis^41^, but also play an important role in antiproliferative, prodifferentiation and anticancer^11^,^12^,^42^,^43^. Taken together, the in-depth studies about vitamin D as a promising anticancer drug need to be urgently carried out.

At present, the mechanisms, account for the beneficial impacts of vitamin D supplement use on clinical outcomes of cancer, can be summarized as follows. One is that vitamin D are involved in various signaling pathways by its role of anticancer active to affect many important molecular events. As mentioned above, they include inhibition of proliferative, angiogenesis, invasion and metastasis, induction of apoptosis and stimulation of differentiation for malignant cells^6^,^10^,^22^. Another recognized mechanism is that vitamin D is also related to immune regulation and inflammation^6^,^22^, which play an important role in cancer pathogenesis and progression. In addition, the other novel mechanisms will be further studied in future.

There are a few limitations in this study. Firstly, because of a retrospective and single-center study, the collected data is somewhat limited. Secondly, vitamin D concentration in blood is not routinely measured among cancer patients in our clinical practice. As a result, the associations of the level of vitamin D with clinical outcomes could not be observed. Thirdly, pre-operative vitamin D supplement use, which may be related to both post-operative vitamin D supplement use and EC outcomes, is unavailable in our study. Therefore, further studies will be needed to verify that our results are not bias or occasional.

In summary, we initially reported that vitamin D supplement use could enhance QOL and prolong DFS of EC. Our study supported that clinician should recommend the use of vitamin D supplementation as an intervention during cancer treatment and recovery phases to improve poor clinical outcomes. Although our results was strongly supported by previous studies, further prospective, well-designed randomized controlled trials with larger samples are necessary to further verify the impacts of vitamin D supplementation on cancer’s clinical outcomes.

## Materials and Methods

### Patient recruitment

Between January 2012 and December 2012, newly diagnosed and pathologically proven EC patients were recruited from the Department of Thoracic Surgery, Qilu Hospital of Shandong University. All patients enrolled in our study had detailed and authentic clinicopathological data. Patients were excluded from the study: (1) if they were treated with chemotherapy and**/**or radiotherapy before surgery to reduce the size of the tumor; (2) if they could not clarify whether vitamin D supplementation was used or not after esophagectomy; (3) if they received other supplementations post operative in addition to vitamin D supplement; (4) if they were lost to follow-up. This study was approved by Ethics Committee of Qilu Hospital of Shandong University. Informed consent was obtained from all the patients. All data has been anonymized and de-identified. Moreover, the patient data collection methods were carried out in accordance with the Declaration of Helsinki.

### Data collection

Clinicopathological data were obtained from patients’ medical records. Metastases to lymph nodes resected at surgery were counted and pathologically examined. Major factors known to influence QOL, recurrence and mortality of EC were extracted from medical records and follow-up, including age at diagnosis, gender, education, income, body mass index (BMI), physical activity, sun exposure, smoking, alcohol, dietary habits, past medical history, tumor characteristics, type of surgery and treatment regimen. All tumors were staged on the basis of the American Joint Committee on Cancer staging manual^44^. The cutoff points of alcohol, as well as fruit and vegetable intake were based on the 2011 Chinese Inhabitant Dietary Guideline. The cutoff values of tumor length and the lymph node ratio were in view of previous articles^45^.

### Vitamin D supplementation assessment

The data of vitamin D supplement use were ascertained through self-report. Each patient or their kin was asked as follows: (1) Did patient take regularly vitamin D supplementation after esophagectomy during EC treatment and recovery phases? (2) If did, what’s the frequency and dose of vitamin D supplementation? (3) What’s the duration of taking it? (4) What’s the brand of vitamin D supplementation? On the basis of these collected data, we finally identified two groups of patients for comparison**—**those who received vitamin D supplementation after surgery during EC treatment and recovery phases (vitamin D supplementation users group) and those who did not use it (vitamin D supplementation non-users group).

### QOL assessment

EC survivors’ QOL were ascertained by using the cancer-specific European Organization for Research and Treatment of Cancer Quality of Life Questionnaire C30 (EORTC QLQ-C30) and oesophagus-specific module EORTC QLQ-OES18 at diagnosis and 24 months after surgery. Both the two questionnaires are well validated and their utilities among EC patients have been previously described^46^,^47^. The EORTC QLQ-C30 comprises one global health scale, five function scales (physical, role, emotional, cognitive and social), three symptom scales (fatigue, pain and nausea**/**vomiting), and six single items (dyspnoea, insomnia, appetite loss, constipation, diarrhea and financial difficulties). The EORTC QLQ-OES18 contains four symptom scales (dysphagia, eating difficulties, reflux and esophageal pain) and six single items (trouble swallowing saliva, choking when swallowing, dry mouth, trouble with taste, trouble with cough and trouble with talking). Each item in both questionnaires, except for the global health scale which has seven responses ranging from “very poor” to “excellent”, have four response categories: “not at all”, “a little”, “quite a bit” and “very much”. Patients’ responses were converted to a score of 0–100 in accordance with the EORTC scoring manual^46^, with higher scores reflecting better QOL in function scales and global health scale, whereas higher scores in symptom scales and items representing more serious symptoms.

### Follow-up and survival assessment

Patients were informed routinely examined in our outpatient clinics every 3 months for the first 2 years post operative and every 6 months interval or until death thereafter. Physical examination, laboratory tests, barium meal fluoroscopy, esophagoscopy, computed tomography scans and other examinations as it fits were included in the follow-up assessments. In brief, the condition of patients’ recurrence or death can be timely obtained through a combination of follow-up and medical record review. The follow-up end point was death or January 2016.

### Statistical analysis

Baseline characteristics of patients were presented as mean ± standard deviation or proportion by postoperative vitamin D supplementation status (users versus non-users). The Student’s t test or Mann-Whitney U test where appropriate was used to evaluate continuous variables. Categorical variables were assessed by using the Pearson’s chi square test or Fisher’s exact test. The primary end point of this study was the QOL of EC survivors at 24 months post operative. GEEs were utilized to analysis the association of QOL with vitamin D supplement use. The secondary end points were 3-year DFS and 3-year OS. DFS was calculated from the date of surgery to the first date of tumor recurrence. OS was defined as the time from the date of surgery to the date of death or to last follow-up. Kaplan**-**Meier curves and log**-**rank tests were used for survival analyses. The Cox regression model was used in the univariate and multivariate analyses. The variables having statistically significant in the univariate analysis were selected into the multivariable analysis. All *p* values were two-sided and *p* < 0.05 indicated statistically significant.

All data were performed with the Statistical Package for Social Science program (SPSS for Windows, version 17.0, SPSS Inc., Chicago, IL).

## Additional Information

**How to cite this article**: Wang, L. *et al*. Longitudinal, observational study on associations between postoperative nutritional vitamin D supplementation and clinical outcomes in esophageal cancer patients undergoing esophagectomy. *Sci. Rep.* **6**, 38962; doi: 10.1038/srep38962 (2016).

**Publisher’s note:** Springer Nature remains neutral with regard to jurisdictional claims in published maps and institutional affiliations.

## Figures and Tables

**Table 1:** Patients’ characteristics.

Characteristics	Postoperative vitamin D supplementation		*p* value
	Users (n = 49)	Non-users (n = 254)	
Age (years)	61.694 ± 6.884	64.886 ± 7.552	0.006*
Gender (M:F)	40:9	212:42	0.754
Education			0.008*
High school and below	35 (71.429%)	220 (86.614%)	
Some college and above	14 (28.571%)	34 (13.386%)	
Income (RMB/month)			0.003*
<1000	12 (24.490%)	105 (41.339%)	
1000~2000	10 (20.408%)	72 (28.346%)	
>2000	27 (55.102%)	77 (30.315%)	
BMI (kg/m^2^)	21.153 ± 3.599	21.576 ± 2.971	0.329
Physical activity (h/week)	16.764 ± 5.698	14.500 ± 5.807	0.013*
Sun exposure (days/week)^a^			0.658
<7	12 (24.490%)	70 (27.559%)	
=7	37 (75.510%)	184 (72.441%)	
Smoking (pack-year)			0.003*
<20	38 (77.551%)	138 (54.331%)	
≥20	11 (22.449%)	116 (45.669%)	
Alcohol (kg/day)			0.142
≤0.025	38 (77.551%)	170 (66.929%)	
>0.025	11 (22.449%)	84 (33.071%)	
Fruit and vegetable intake^b^			0.519
≥300 g/day & ≥200 g/day	26 (53.061%)	122 (48.031%)	
Fat intake			0.020*
High	10 (20.408%)	54 (21.260%)	
Medium	15 (30.612%)	125 (49.213%)	
Low	24 (48.980%)	75 (29.527%)	
Past medical history			
HBP	8 (16.327%)	51 (20.079%)	0.544
CAD	5 (10.204%)	15 (5.906%)	0.426
Diabetes	6 (12.245%)	7 (2.756%)	0.009*
Tumor information			
Pathology			0.897
SCC	46 (93.878%)	234 (92.126%)	
AC	3 (6.122%)	20 (7.874%)	
Histological grade			0.252
Well	4 (8.163%)	43 (16.929%)	
Moderately	19 (38.776%)	99 (38.976%)	
Poorly/undifferentiated	26 (53.061%)	112 (44.095%)	
Location			0.143
Cervical/upper/middle	25 (51.020%)	158 (62.205%)	
Low	24 (48.980%)	96 (37.795%)	
Length <3 cm	22 (44.900%)	105 (41.339%)	0.644
T category			0.696
0	2 (4.082%)	6 (2.362%)	
1	7 (14.286%)	36 (14.173%)	
2	15 (30.612%)	77 (30.315%)	
3	25 (51.020%)	126 (49.606%)	
4	0 (0%)	9 (3.544%)	
TNM stage			0.884
0/I/II	32 (65.306%)	158 (62.205%)	
III/IV	17 (34.694%)	96 (37.795%)	
Lymph node metastasis	23 (46.939%)	113 (44.488%)	0.752
No. of metastatic lymph nodes	1.102 ± 1.862	1.591 ± 3.012	0.918
Ratio of lymph node <0.2	41 (83.673%)	202 (79.528%)	0.505
Type of surgery			0.966
*L*-thoracic esophagectomy	40 (81.633%)	208 (81.890%)	
*R*-thoracic esophagectomy	9 (18.367%)	46 (18.110%)	
Treatment regimen			0.179
S	21 (42.857%)	153 (60.236%)	
S plus postoperative R	2 (4.082%)	23 (9.055%)	
S plus postoperative C	12 (24.490%)	41 (16.142%)	
S plus postoperative CRT	14 (28.571%)	37 (14.567%)	

M, male; F, female; BMI, body mass index; HBP, high blood pressure; CAD, coronary artery disease; SCC, squamous cell carcinoma; AC, adenocarcinoma; *L*, left; *R*, right; S, surgery; R, radiotherapy; C, chemotherapy; CRT, chemoradiotherapy. ^a^Sun exposure was defined as the number of days per week on which at least half an hour was spent outside. ^b^The cutoff values of fruit and vegetable are ≥300 g/day and ≥200 g/day, respectively. *p < 0.05

**Table 2:** Multivariable-adjusted association between postoperative vitamin D supplement use and QOL assessed by EORTC QLQ-C30 and QLQ-OES18 over 24-month follow up.

	*β* coefficient	95% CI	*p* value
**QLQ-C30**			
Global Health	−2.985	−5.880, −0.089	0.043*
** Functional Scales**			
Physical Functioning	−3.640	−6.085, −1.196	0.004*
Role Functioning	−0.449	−4.070, 3.173	0.808
Emotional Functioning	−2.621	−7.336, 2.095	0.276
Cognitive Functioning	−2.017	−9.191, 5.158	0.582
Social Functioning	−6.347	−11.178, −1.516	0.010*
** Symptom Scales**			
Fatigue	6.110	0.531, 11.689	0.032*
Nausea/vomiting	−0.717	−4.476, 3.042	0.709
Pain	0.894	−4.579, 6.368	0.749
Dyspnoea	−1.099	−4.777, 2.580	0.558
Insomnia	3.456	−0.827, 7.739	0.114
Appetite loss	10.435	1.107, 19.763	0.028*
Constipation	3.131	−7.324, 13.585	0.557
Diarrhea	−0.380	−4.160, 3.401	0.844
Financial difficulties	1.972	−3.551, 7.495	0.484
***QLQ-OES18***			
Dysphagia Scale	−0.614	−5.509, 4.281	0.806
Eating Scale	5.365	0.876, 9.853	0.019*
Reflux Scale	5.341	−3.428, 14.109	0.233
Pain Scale	−4.160	−8.924, 0.604	0.087
Trouble swallowing saliva	3.151	−1.916, 8.218	0.223
Choking when swallowing	−4.470	−9.737, 0.796	0.096
Dry mouth	2.206	−1.907, 6.318	0.293
Trouble with taste	8.491	0.882, 16.100	0.029*
Trouble with coughing	2.470	−1.410, 6.349	0.212
Trouble with talking	0.425	−3.681, 4.530	0.839

CI, confidence interval. *p < 0.05.

**Table 3:** Univariate analysis of factors associated with OS and DFS.

	OS			DFS		
	*p* value	HR	95% CI	*p* value	HR	95% CI
Age	0.160	1.015	0.994-1.036	0.075	1.019	0.998–1.039
Male	0.023*	0.566	0.346–0.925	0.619	0.900	0.595–1.361
Education	0.777	1.063	0.698–1.619	0.250	1.262	0.849–1.877
High school and below						
Income						
<1000 RMB/month	0.336	Ref.		0.571	Ref.	
1000~2000	0.152	1.330	0.900–1.964	0.957	0.989	0.664–1.474
>2000	0.320	1.208	0.833–1.753	0.356	1.183	0.828–1.690
BMI	0.558	0.985	0.937–1.036	0.906	1.003	0.954–1.054
Physical activity	<0.001*	0.942	0.916–0.968	<0.001*	0.946	0.920–0.972
Sun exposure	0.691	1.074	0.754–1.530	0.907	0.980	0.696–1.379
<7 days/week						
Smoking	0.991	1.002	0.730–1.375	0.626	1.081	0.791–1.476
<20 pack-year						
Alcohol	0.299	0.831	0.587–1.178	0.310	0.837	0.593–1.180
≤0.025 kg/d						
Fruit and vegetable	0.318	0.852	0.622–1.167	0.364	0.866	0.635–1.181
≥300 g/d &≥200 g/d						
Fat intake						
High	0.526	Ref.		0.397	Ref.	
Medium	0.354	1.218	0.803–1.848	0.700	0.926	0.626–1.370
Low	0.903	1.028	0.656–1.613	0.205	0.760	0.497–1.161
Past medical history						
HBP	0.672	1.089	0.735–1.613	0.409	0.840	0.556–1.271
CAD	0.687	0.877	0.462–1.664	0.855	0.944	0.512–1.742
Diabetes	0.981	0.991	0.464–2.114	0.991	0.996	0.467–2.124
Tumor information						
Pathology	0.026*	1.798	1.071–3.017	0.003*	2.172	1.311–3.598
SCC						
Histological grade						
Well	0.118	Ref.		0.452	Ref.	
Moderately	0.299	1.316	0.784–2.209	0.762	0.930	0.582–1.487
Poorly/undifferentiated	0.054	1.636	0.992–2.699	0.536	1.153	0.735–1.809
Location	0.792	0.958	0.696–1.319	0.548	0.908	0.662–1.245
Cervical/upper/middle						
Length<3 cm	<0.001*	0.430	0.305–0.607	<0.001*	0.540	0.391–0.747
T category						
0	0.016*	0.076	0.009–0.615	0.056	0.259	0.065–1.038
1	<0.001*	0.115	0.041–0.317	0.001*	0.207	0.078–0.546
2	0.013*	0.363	0.163–0.806	0.103	0.494	0.211–1.153
3	0.118	0.542	0.251–1.169	0.238	0.607	0.265–1.391
4	<0.001*	Ref.		0.002*	Ref.	
TNM stage	<0.001*	2.905	2.116–3.990	<0.001*	2.452	1.793–3.355
0/I/II						
Lymph node metastasis	<0.001*	2.771	2.002–3.836	<0.001*	2.093	1.531–2.860
No. of metastatic lymph nodes	<0.001*	1.131	1.089–1.174	<0.001*	1.164	1.113–1.218
Ratio of lymph node<0.2	<0.001*	0.393	0.279–0.553	<0.001*	0.418	0.293–0.597
Type of surgery						
Left-thoracic esophagectomy	0.305	1.226	0.831–1.807	0.263	1.251	0.845–1.852
Treatment regimen						
S	0.013*	Ref.		0.004*	Ref.	
S plus postoperative R	0.003*	2.129	1.287–3.522	0.042*	1.774	1.020–3.085
S plus postoperative C	0.155	1.358	0.891–2.070	0.098	1.419	0.937–2.149
S plus postoperative CRT	0.040*	1.555	1.020–2.370	0.001*	1.976	1.327–2.942
Vitamin D supplementation	0.308	0.795	0.511–1.237	0.035*	0.611	0.386–0.966

HR, hazard ratio; CI, confidence interval; BMI, body mass index; HBP, high blood pressure; CAD, coronary artery disease; SCC, squamous cell carcinoma; S, surgery; R, radiotherapy; C, chemotherapy; CRT, chemoradiotherapy. *p < 0.05.

**Table 4:** Multivariate analysis of factors associated with OS and DFS.

	OS			DFS		
	*p* value	HR	95% CI	*p* value	HR	95% CI
Male	0.132	0.675	0.404–1.126	—	—	—
Physical activity	0.004*	0.957	0.928–0.986	<0.001*	0.949	0.922–0.978
Pathological type	0.589	1.169	0.663–2.061	0.059	1.682	0.981–2.883
SCC						
Length<3 cm	0.096	0.729	0.503–1.058	0.095	0.733	0.509–1.056
T category						
0	0.186	0.230	0.026–2.034	0.935	1.066	0.227–5.011
1	0.022*	0.253	0.078–0.817	0.530	0.686	0.212–2.220
2	0.304	0.606	0.233–1.576	0.630	1.325	0.459–3.822
3	0.423	0.703	0.297–1.665	0.764	1.160	0.441–3.049
4	0.094	Ref.		0.376	Ref.	
TNM stage	0.968	1.012	0.558–1.836	0.158	1.574	0.839–2.955
0/I/II						
Lymph node metastasis	0.097	1.619	0.917–2.856	0.786	0.922	0.514–1.655
No. of metastatic lymph nodes	0.246	1.040	0.973–1.111	0.059	1.076	0.997–1.161
Ratio of lymph node<0.2	0.406	0.812	0.496–1.327	0.993	1.002	0.593–1.695
Treatment regimen						
S	0.480	Ref.		0.158	Ref	
S plus postoperative R	0.142	1.475	0.878–2.480	0.323	1.336	0.752–2.375
S plus postoperative C	0.831	0.952	0.608–1.493	0.339	1.236	0.800–1.910
S plus postoperative CRT	0.964	1.010	0.650–1.571	0.026*	1.628	1.060–2.500
Vitamin D supplementation	—	—	—	0.040*	0.610	0.381–0.978

HR, hazard ratio; CI, confidence interval; SCC, squamous cell carcinoma; S, surgery; R, radiotherapy; C, chemotherapy; CRT, chemoradiotherapy.

*p < 0.05
